# Load-Bearing Increase and Damage Progression in CF/PEEK Thermoplastic Laminates Under Repeated Low-Velocity Impacts

**DOI:** 10.3390/polym18040509

**Published:** 2026-02-19

**Authors:** Jiezheng Qiu, Chunxing Hu, Zhonghai Xu

**Affiliations:** National Key Laboratory of Science and Technology on Advanced Composites in Special Environments, Harbin Institute of Technology, Harbin 150080, China

**Keywords:** thermoplastic composites, low-velocity impacts, energy absorption, nondestructive testing

## Abstract

Carbon fiber-reinforced polyetheretherketone (CF/PEEK) thermoplastic composites are increasingly applied in aerospace structures due to their outstanding mechanical and thermal properties. However, their strengthening mechanism and damage evolution under repeated low-velocity impacts remains inadequately explored. This study systematically investigates the mechanical response and failure mechanisms of CF/PEEK laminates subjected to sequential single and second impacts at energy levels of 10 J, 20 J, and 30 J. Through comprehensive analysis of impact parameters (peak load, energy absorption, residual displacement), optical microscopy and ultrasonic C-scan, this study reveals that the load-bearing increase under repeated low-velocity impacts results from the combined effects of multiple mechanisms, including matrix plastic deformation, local compaction, matrix damage, and interlaminar failure. Under initial impacts, laminates exhibit high load-bearing capacity and energy dissipation, which are dominated by plastic deformation and matrix failure at 10 J and 20 J, whereas the 30 J impact causes pronounced fiber failure. An anomalous increase in peak load is observed during secondary impacts, which is attributed to matrix compaction-induced strengthening resulting from the initial impact. Optical microscopy and C-scan quantification demonstrate that, while the initial impact induces compaction-related strengthening, it also causes internal damage, which leads to aggravated damage evolution during the subsequent impact. The findings provide fundamental insights into damage accumulation in thermoplastic composites and directly inform impact-resistant design strategies.

## 1. Introduction

CF/PEEK composites have been widely used in aerospace, rail transportation, and high-end equipment manufacturing due to the high specific strength, high specific stiffness, excellent thermal resistance, and chemical stability of the material [1,2]. Compared with conventional thermosetting composites, CF/PEEK exhibits superior toughness and weldability, and demonstrates enhanced energy absorption capacity and impact resistance under dynamic loading conditions [3,4,5,6]. Under single impact conditions, CF/PEEK exhibits a strong resistance to crack growth and interlaminar delamination, with failure characteristics that markedly differ from thermoset composites [7,8,9]. However, in actual service environments, composite structures are frequently subjected to complex loading scenarios involving repeated low-velocity impacts, where the accumulation of damage poses a potential threat to both mechanical performance and structural integrity [10,11].

Extensive research has been conducted to investigate the repeated low-velocity impact behavior of composite laminates. Zhou et al. [12] systematically investigated the mechanical response and damage evolution characteristics of CFRP laminates subjected to double-impact loading at two distinct locations. Lyu et al. [13] systematically investigated the influence of different distances between impact positions on the post-impact compressive damage and failure behavior of carbon/glass fiber-reinforced composites under multiple low-velocity impacts. Liao et al. [14] investigated the low-velocity multiple impact response and damage mechanisms of composite laminates under varying distances between impact positions. Zhou et al. [15] systematically investigated the dynamic response and damage evolution mechanisms of cross-ply laminates under repeated low-velocity impacts. These studies revealed that the spacing between impact sites significantly influences the coupling effect and damage morphology between successive impacts. The results showed that under low-energy impacts, the second impact exhibited a stiffness enhancement effect, whereas under high-energy impacts, the initial damage weakened the load-bearing capacity of the structure, leading to more severe damage. Sadighi et al. [16] conducted a comprehensive review of the damage evolution behavior of fiber-reinforced polymers under repeated and repeated low-velocity impacts. A unified terminology framework was proposed, along with the classification of four main research pathways in impact fatigue. The review highlighted that, compared with single impacts, repeated impacts can induce severe progressive damage even at low energy levels, typically exhibiting a three-stage pattern: rapid damage accumulation in the initial stage, a stable plateau in the intermediate stage, and sudden failure in the final stage.

The repeated impact behavior of thermoplastic composites has also attracted increasing attention from researchers [16,17,18]. Hu et al. [19] conducted a systematic study on the mechanical response and damage interference mechanisms of CF/PEEK thermoplastic composites under double-location low-velocity impacts. The results demonstrated that the coupling between impact spacing and impact energy governs the degree of interference between the two impacts. When the spacing was small, higher impact energy led to more pronounced damage superposition effects, including phenomena such as fiber breakage and crack coalescence. Li et al. [20] conducted double-location low-velocity impact and compression-after-impact tests on glass fiber-reinforced laminates with three typical layup configurations: cross-ply, ±45° angled, and quasi-isotropic. The study systematically revealed the influence of stacking sequence on damage interference effects and residual mechanical performance. Dogan et al. [21] systematically investigated the low-velocity impact response of thermoset and thermoplastic composites, with particular focus on how impactor shape, impact repetition, matrix type, and fiber volume fraction influence the perforation threshold and impact resistance. Psarras et al. [22] systematically analyzed the damage tolerance of carbon fiber-reinforced composite plates under multi-site low-velocity impacts, evaluating the mechanical response of plates with different thicknesses subjected to repeated impacts. Reis et al. [23] conducted a study on the repeated low-velocity impact behavior of thermoplastic composite laminates under different energy levels. The results indicated that, under multiple impacts, the fatigue life of the specimens was inversely proportional to the impact energy. In studies on the repeated-impact behavior of thermoset composites, many researchers have reported different phenomena. Morais et al. [24] investigated repeated low-energy impacts on carbon/epoxy laminates and analyzed damage accumulation using load–displacement curves and impact histories. The results showed that, as the number of impacts increased, the initial slope of the curve decreased, which indicates a progressive reduction in laminate stiffness. Meanwhile, the impact duration increased, which reflects an increase in compliance caused by local damage. Liao et al. [25] conducted comparative tests on repeated low-velocity impacts of laminated composites and reported that the first impact induced an initial stiffness change with a delamination initiation threshold. During repeated impacts, this threshold-related feature was no longer observed, and the load curve became smoother. Rezasefat et al. [26] studied the repeated-impact responses of glass/aramid–epoxy composites at 19 J and 37 J. The results indicated that increasing the number of impacts led to a reduction in flexural stiffness; however, the stiffness degradation depended on the lay-up. Some aramid-containing lay-ups exhibited negligible stiffness loss after the early impacts, whereas the high-stiffness glass-dominated lay-ups showed pronounced degradation after multiple impacts.

For the prediction of impact behavior, several researchers have also conducted relevant studies. Zhao et al. [27] proposed a machine learning approach to predict the compression-after-impact strength of carbon/glass hybrid laminates subjected to repeated low-velocity impacts. The study revealed that the peak load during the second impact was higher than that of the initial impact, while the subsequent impacts exhibited a stabilized force response. In contrast, the energy absorption capacity dropped significantly after the first impact, indicating pronounced stiffness degradation and a trend toward damage saturation. Zhou et al. [28] developed a user-defined subroutine that integrates the Hou failure criterion with a cohesive zone model to predict the dynamic response and damage mechanisms of composite laminates under both single and repeated low-velocity impacts. The study found that as the laminate thickness increased, impact response parameters—including peak load, displacement, and energy dissipation—exhibited linear trends. Moreover, the dominant damage mode gradually shifted from intralaminar fiber/matrix failure to delamination-dominated behavior. Rezasefat et al. [29] developed a VUMAT with the Puck criterion and nonlinear shear, coupled with cohesive elements to model repeated low-velocity impacts in CFRP laminates. The results indicated that the majority of damage was induced during the first impact, while the energy absorption capacity in subsequent impacts tended to stabilize, reflecting stiffness degradation and a trend toward damage saturation. Fang et al. [30] investigated the damage evolution mechanisms of carbon fiber/epoxy composite laminates under repeated low-velocity impacts through a combined experimental and numerical approach. The study revealed that matrix tensile failure was the predominant damage mode, and the second impact contributed most significantly to the propagation of damage.

Numerous studies have shown that composite materials may exhibit a certain degree of strengthening effect under secondary impact. However, current research lacks systematic understanding of this phenomenon. In particular, studies on the strengthening effect of thermoplastic composites under secondary impact are even more limited, and the underlying mechanisms have yet to be clearly explained. This study focuses on the damage evolution and delamination behavior of CF/PEEK subjected to secondary low-velocity impacts. By combining damage morphology observation, energy absorption analysis, and quantitative evaluation of delamination, the study focuses on understanding the relationship between impact energy, energy dissipation, and damage growth. The results will offer theoretical support for the reliability assessment and design improvement of CF/PEEK under repeated impact conditions.

## 2. Material Preparation and Experiments

### 2.1. Composite Material and Preparation

The composite laminates used in this study were fabricated from CF/PEEK prepreg supplied by Jiangsu Hengbo Composite Materials Co., Ltd. (Danyang, China). Following the ASTM D7136 standard for low-velocity impact testing of composites. The laminates were prepared with dimensions of 150 mm × 100 mm and a thickness of 2.4 mm [31,32]. The stacking sequence was defined as [0,90]_2s_ for 16 plies [33]. The fabrication process was carried out using a hot-press molding technique to ensure adequate interlaminar bonding quality and thickness uniformity. The manufacturing procedure was provided by the prepreg manufacturer and is consistent with our previous studies [34].

### 2.2. Low-Velocity Impact Experiments

**Figure 1:**
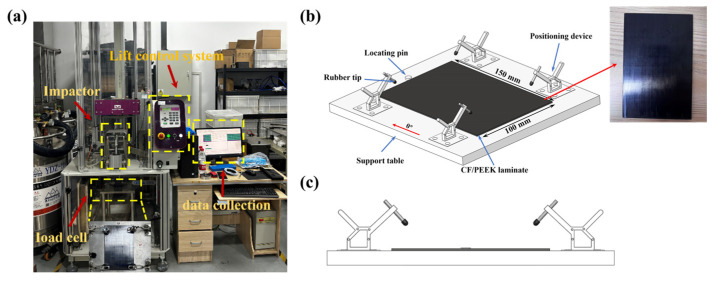
Low-velocity impact experiment system: (**a**) experimental setup; (**b**) fixture configuration; (**c**) sample mounting.

The low-velocity impact experiments were conducted by using a drop-weight impact system in accordance with the ASTM D7136 standard. As shown in Figure 1, each sample was fixed within the experimental fixture using a four-edge simply supported configuration to simulate typical boundary constraints encountered in actual structures. A locating pin was used during fixture setup to ensure that the impact occurred at the center of the specimen. Before each impact, full contact between the sample and the fixture was verified. A hemispherical steel impactor with a diameter of 16 mm and a mass of 5.4 kg was used to apply impact loading at the center of the sample [35]. Single and repeated impact tests were conducted on thermoplastic composite laminates at 10 J, 20 J, and 30 J, and two specimens were tested for each energy level.

Because direct displacement measurement may introduce relatively large errors, the displacement and velocity of the impactor in the impact tests were obtained based on the force signal measured by the load cell. Different impact energy levels were achieved by adjusting the initial velocity of the impactor. During each experiment, the impact force–time history and energy absorption characteristics were recorded to evaluate the mechanical response of the laminates under repeated impacts. The system continuously collected force–time data, and the variation in impactor velocity and energy dissipation was further determined based on the initial velocity and accelerometer measurements. The impactor velocity was obtained by time integration of the acceleration derived from the measured force and the known impactor mass. The impactor displacement was then calculated by integrating the velocity over time. The absorbed (dissipated) energy was finally determined from the reduction in kinetic energy relative to the initial value, as described in Equations (1)–(3) [36].

(1) $$\nu_{i}(t)=\nu_{0}-\int_{0}^{t}\frac{F(t)}{m}\;dt$$

(2) $$S(t)=\int_{0}^{t}\left[\nu_{0}-\int_{0}^{t}\frac{F(t)}{m}\;dt\right]\;dt$$

(3) $$E_{a}(t)=E_{k0}-\frac{1}{2}m{\left[\nu_{0}-\frac{1}{m}\int_{0}^{t}F(t)\;dt\right]}^{2}$$

where *m* and *v*_0_ represent the mass and initial velocity of the impactor, respectively; *E_k_*_0_ denotes the initial kinetic energy of the impactor; and *E_a_*(*t*) refers to the energy absorbed by the sample at any given time *t*. When *E_a_*(*t*) approaches a constant value over time, this steady-state value is regarded as the total energy absorbed by the sample during the entire impact process, which is used to characterize the energy absorption capacity of the CF/PEEK laminate. *F*(*t*) denotes the impact force as a function of time during the impact event; *S*(*t*) is defined as the displacement of the impactor as a function of time; and *dt* is the differential of time, which represents an infinitesimal time increment used in the time integration.

### 2.3. Morphology Characterization and Nondestructive Scanning

**Figure 2:**
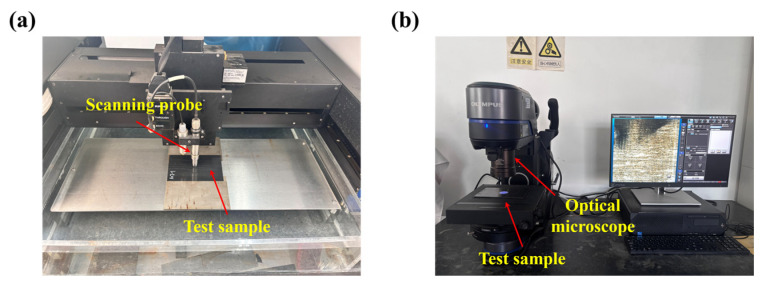
Morphology characterization and nondestructive scanning setup: (**a**) ultrasonic C-scan; (**b**) optical microscope.

To systematically investigate the damage evolution of CF/PEEK laminates under repeated impacts, each sample was subjected to sequential single and secondary impact loading, followed by nondestructive evaluation and microscopic observation after each impact (Figure 2). After the first impact, ultrasonic C-scan and optical microscopy were employed to assess damage. The C-scan technique, based on pulse–echo imaging, was used to obtain two-dimensional images of interlaminar delamination and debonding regions. A water-coupling method was adopted to enhance detection accuracy. In parallel, the impacted sample was prepared and examined under an optical microscope to observe microscopic damage features, including matrix cracking, delamination boundaries, and localized fiber breakage [37,38]. The burrs generated around the impact region after impact were carefully removed to avoid interference during imaging. The specimen was then placed flat on the microscope stage, and the objective lens was adjusted to focus on the impact-damaged region for observation.

After completing the first round of inspection, the same sample was subjected to a second impact, followed by repeated C-scan and microscopic observation procedures. By comparing the damage extent and microstructural changes before and after the second impact, the cumulative damage behavior and damage tolerance characteristics of CF/PEEK laminates under repeated impacts were revealed.

## 3. Results and Discussion

To study how the first and second impacts affect the mechanical response and damage of CF/PEEK laminates, this study extracted and comparatively analyzed the impact force–time response curves, surface morphologies of the impacted regions, and delamination distributions under different impact energy levels. By correlating mechanical responses with observed damage morphologies, the evolution of mechanical behavior and the mechanisms of damage propagation under single and repeated impacts were revealed.

### 3.1. Low-Velocity Impact Response

#### 3.1.1. Time–Force Response

**Figure 3:**
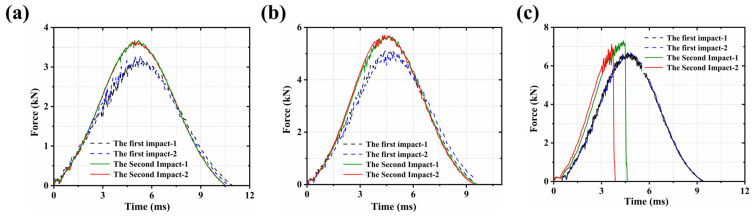
Time–force curves of the first and second impacts: (**a**) 10 J; (**b**) 20 J; (**c**) 30 J.

After experiencing the first impact, the same sample was subjected to a second impact with the same energy level, and the corresponding force–time response curves were extracted. Unlike conventional thermosetting composites, which typically exhibit a decrease in peak load under repeated impacts, the CF/PEEK laminates in this study showed an increase in peak load during the second impact at all three energy levels (10 J, 20 J, and 30 J) [12]. As shown in Figure 3, the peak loads at each energy level increased to different extents, indicating that the structural strength was not significantly degraded by the initial localized damage. On the contrary, n load-bearing increase was observed during the second impact.

This unusual behavior can be attributed to the combined effects of thermoplastic matrix plastic deformation, local compaction, and matrix deformation and damage in CF/PEEK laminates. During the first impact, part of the impact energy was dissipated through matrix plastic flow and micro-scale damage, resulting in the formation of locally densified regions. This process did not cause severe interlaminar failure; instead, it led to a more stable local structure due to compaction, thereby contributing to a higher load-bearing capacity during the second impact [3,12,38,39].

However, the load-bearing increase induced by the initial impact is clearly limited, as it results only from temporary local densification and plastic adjustment of the structure. During the secondary impact, the pre-existing micro-damage regions tend to expand significantly, particularly near the delamination interfaces, where extensive debonding and crack propagation occur. Under lower impact energies (e.g., 10 J and 20 J), although penetration failure did not occur, the force–time curves exhibited a relatively gradual decline after reaching the peak, indicating that the laminates retained a certain level of residual load-bearing capacity. In contrast, under higher impact energy (e.g., 30 J), the secondary impact triggered rapid local structural instability, eventually leading to penetration failure. This was reflected by a sharp drop in the load curve, signifying that the structure had lost its load-bearing capability, as shown in Figure 3c.

In addition, the peak loads and corresponding changes between the first and second impacts of CF/PEEK laminates under three different impact energy levels (10 J, 20 J, and 30 J) were compared, as shown in Table 1. It could be observed that, at all energy levels, the peak load during the second impact was higher than that of the first impact, with increases of 0.405 kN, 0.789 kN, and 0.545 kN, corresponding to relative increases of 12.64%, 16.12%, and 8.17%, respectively.

These results indicated that CF/PEEK laminates possessed strong damage tolerance and plastic energy absorption capability under low to moderate impact energy. However, under repeated high-energy impacts, the laminates were prone to rapid damage accumulation and faced an increased risk of structural failure.

#### 3.1.2. Time–Energy Response

To further investigate the energy absorption mechanism of CF/PEEK laminates under repeated impacts, time–energy response curves were extracted. These curves provide a direct representation of the material’s ability to absorb and dissipate energy during the impact process.

**Figure 4:**
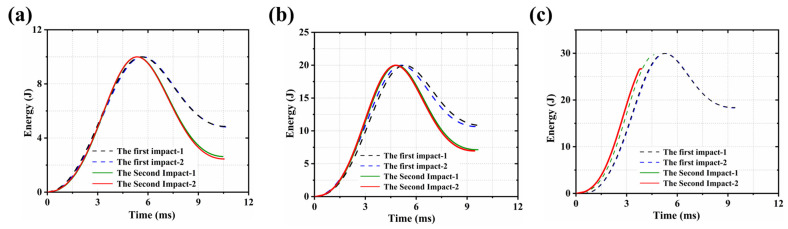
Time–energy curves of the first and second impacts: (**a**) 10 J; (**b**) 20 J; (**c**) 30 J.

As shown in Figure 4, during the first impact, CF/PEEK laminates exhibited excellent energy absorption capacity and favorable impact response under all three impact energy levels (10 J, 20 J, and 30 J). When the impactor contacted the sample, a portion of the impact energy was initially absorbed through elastic deformation of the structure. As the energy input continued, micro-scale damage began to occur in the material, including matrix cracking and interlaminar debonding as early-stage failure modes. With increasing impact displacement, the absorbed energy accumulated rapidly and was dissipated through larger-scale mechanisms such as delamination propagation, plastic deformation, and localized fiber breakage. Eventually, the energy was fully absorbed, and the impactor rebounded. Overall, no penetration failure was observed under any of the three energy levels, indicating good damage tolerance and structural integrity of the laminates.

During the second impact, the energy absorption behavior of CF/PEEK laminates was found to follow a trend similar to the first impact. Rebound was clearly observed at 10 J and 20 J, whereas pronounced perforation occurred under the 30 J secondary impact. However, a noticeable reduction in the total absorbed energy was recorded compared to the first impact. This decrease was primarily attributed to the initial damage introduced during the first loading, which the structural continuity and deformation capacity were weakened. As a result, the laminate was unable to dissipate impact energy effectively. In addition, the loss of energy absorption capacity in regions where plastic yielding or failure had occurred led to a reduction in the effective energy-dissipating area. Furthermore, the damage propagation paths became shorter during the second impact, which allowed energy to be more easily transferred to locally damaged regions. This change in energy distribution triggered rapid local instability rather than gradual dissipation.

As shown in Figure 4 at different impact energy levels, higher impact energy was associated with faster energy absorption. In such cases, the existing damaged regions acted as stress concentrators, which promoted localized penetration failure and accelerated the reduction in energy dissipation capacity. At an impact energy of 30 J, penetration was observed.

These results suggest that CF/PEEK laminates exhibited a pronounced damage accumulation effect under repeated impacts. The ability of dissipate energy was gradually weakened as the number of impacts increased.

In Table 2, the absorbed energies during the first and second impacts of CF/PEEK laminates under different impact energy levels (10 J, 20 J, and 30 J) were compared. The results showed that, at impact energies of 10 J and 20 J, the energy absorbed during the second impact decreased significantly compared to the first impact, with reductions of 2.294 J and 3.685 J, corresponding to decreases of 47.42% and 34.11%, respectively. This indicated that a certain degree of plastic deformation and delamination had already occurred during the first impact, which weakened the laminate’s ability to dissipate energy in the second impact. For the impact energy of 30 J, penetration occurred during the second impact, which caused severe damage and made energy data unavailable. Overall, the trend indicated that with increasing energy in the first impact, the material exhibited greater energy absorption capacity. However, the first impact compromised structural integrity, leading to a significant reduction in energy absorption ability during the second impact.

#### 3.1.3. Displacement–Force Response

The displacement–force response curve is one of the key indicators for evaluating the impact behavior and mechanical performance of materials. It reflects the entire evolution of the load as a function of displacement during impact loading, providing comprehensive insight into the structural stiffness, deformation capacity, and damage progression characteristics.

**Figure 5:**
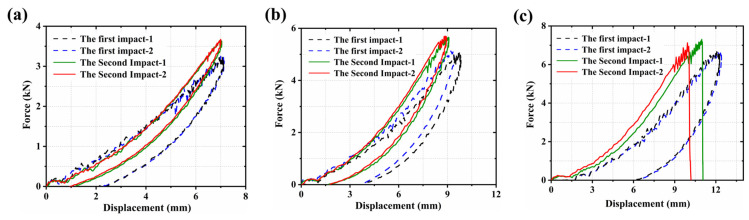
Displacement–force curves of the first and second impacts: (**a**) 10 J; (**b**) 20 J; (**c**) 30 J.

As shown in Figure 5, the load–displacement curves indicated that during the first impact, CF/PEEK laminates exhibited favorable impact resistance under all three impact energy levels. As the impact energy increased, the maximum displacement increased significantly, which suggested that larger structural deformation had occurred under higher energy impacts. In addition, the unloading paths did not return to the initial displacement positions, and the residual displacement increased progressively. This indicated that greater plastic deformation and matrix damage had been introduced under high-energy impacts.

During the second impact, a noticeable change was observed in the characteristics of the load–displacement curves. Compared with the first impact, the maximum displacement decreased at all three energy levels, and this reduction became more pronounced as the impact energy increased. This trend is attributed to a local stiffness increase arising from the combined effects of matrix plastic deformation, local compaction, and matrix damage.

At this stage, the impact energy was primarily absorbed through failure mechanisms such as fiber breakage and interlaminar delamination, rather than through large-scale plastic flow. As a result, the residual deformation after unloading was relatively small, reflecting that the structure had approached its failure limit. This conclusion was further supported by the comparative analysis of post-impact morphologies. When the impact energy reached 30 J, the displacement–force curve exhibited a distinct abnormal feature: a sharp drop at the end of the curve and no further increase in residual deformation. This behavior suggested that penetration failure occurred during the second impact. The impact energy could not be fully dissipated through plastic deformation, leading to structural failure and loss of load-bearing capacity.

In addition, a comparison of the envelope areas under the load–displacement curves from the first and second impacts further demonstrated a reduction in the energy absorption capacity of CF/PEEK laminates after experiencing an initial impact. During the second impact, the area under the curve decreased noticeably, indicating a decline in the material’s ability to dissipate energy under repeated loading conditions. This trend was consistent with the time–energy response patterns shown in Figure 2, further confirming the presence of significant damage accumulation under repeated impacts. Such accumulation was manifested by the shrinkage of plastic energy dissipation regions and the degradation of structural integrity.

### 3.2. Morphology Analysis

To further elucidate the damage evolution mechanisms of CF/PEEK laminates under repeated impacts, morphology characterization was conducted by using optical microscopy on the samples after the first and second impacts. The observations were compared with the corresponding mechanical response curves for comprehensive analysis.

**Figure 6:**
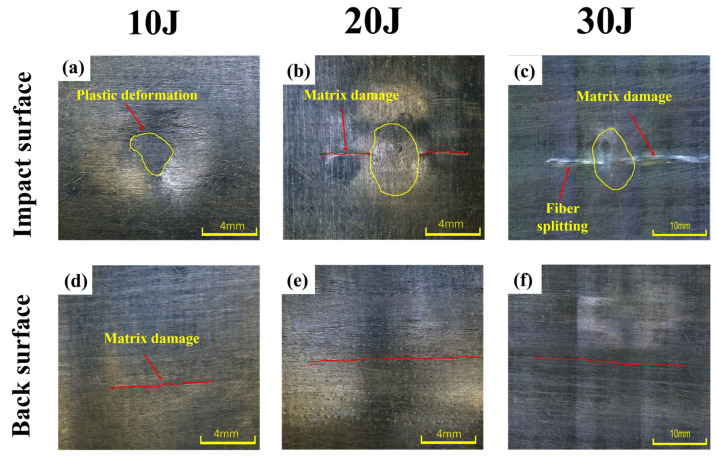
Typical damage morphology of CF/PEEK laminates after the first impact: (**a**–**c**) impacted surface at 10 J, 20 J, and 30 J; (**d**–**f**) corresponding back surface at 10 J, 20 J, and 30 J.

The typical microscopic damage morphology of CF/PEEK laminates after impact testing is shown in Figure 6. Figure 6a–c represent the impacted surfaces of the samples, while Figure 6d–f show the corresponding back-side regions.

In Figure 6a, a distinct plastic indentation region was observed, with a smooth contour and well-defined boundary. This indicates that the impact energy was mainly absorbed through matrix yielding and compaction, without causing severe structural damage. In Figure 6d, only a fine matrix surface crack was detected on the back side, which suggests that the impact energy was not sufficient to trigger perforation and that the overall structural integrity remained intact.

When the impact energy increased to 20 J, more complex damage features were observed in Figure 6b,e. In Figure 6b, matrix cracking and slight interfacial delamination appeared on the impacted surface, indicating that energy transmission had caused coupled failure of the matrix and interface. The back-side image in Figure 6e showed a longer surface crack compared to the 10 J case, exhibiting a transverse, through-thickness pattern, which suggested that the structure had entered a clear damage propagation stage under this energy level.

At 30 J impact energy, Figure 6c revealed a mixed failure mode on the impacted surface, characterized by matrix cracking combined with fiber splitting, indicating that the impact energy had propagated into the reinforcement phase and caused damage to the load-bearing fiber layers. The corresponding back-side image in Figure 6f showed a wider and more continuous matrix crack, suggesting that the energy had not only propagated fully through the laminate thickness but also induced severe failure on the rear surface, with a clear trend toward penetration.

**Figure 7:**
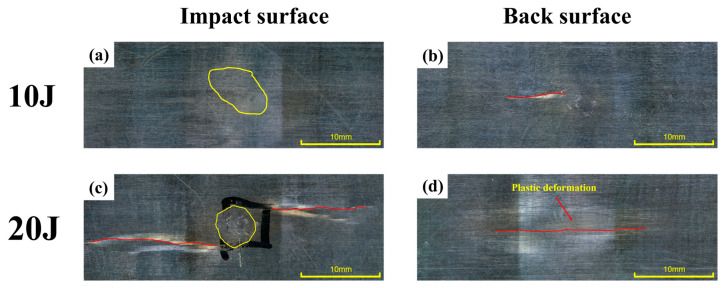
Typical damage morphology of CF/PEEK laminates after the second impact: (**a**,**b**) impacted and back surfaces at 10 J; (**c**,**d**) impacted and back surfaces at 20 J.

Compared with the first impact, more severe damage was observed under the same energy levels during the second impact at 10 J and 20 J. Both the impacted and back surfaces exhibited more extensive matrix cracking and signs of penetration. Figure 7a,b show the microscopic morphologies of the impacted surface and the corresponding back surface of the CF/PEEK laminate after the second impact at 10 J. In Figure 7a, a certain degree of plastic deformation and indentation residue was observed in the local matrix region, but the overall surface morphology remained relatively intact with minimal cracking, indicating that the specimen retained some load-bearing and energy-absorbing capacity after the first impact. Figure 7b shows that back-side damage was minimal, with only slight surface disturbance in the matrix and no observable through-thickness cracks or delamination. This suggests that the second impact at 10 J did not cause significant penetration damage along the thickness direction. Figure 7c,d illustrate the surface and back-side damage after the second impact at 20 J, with clearly intensified damage features. In Figure 7c, a distinct matrix crack propagation zone was observed on the impacted surface, accompanied by signs of surface matrix delamination. This indicates that the initial damage area had further evolved under the second impact, and the structural load-bearing capacity had been significantly reduced. Figure 7d shows apparent matrix failure on the back surface, suggesting that the impact energy had propagated through the laminate thickness and caused local matrix failure on the rear side. Compared with the first impact, the damage exhibited a clear worsening trend; however, extensive fiber breakage was not observed, indicating that the structure still retained a certain level of load-bearing capacity. Overall, the damage behavior under both impact energies was significantly more severe than that observed during the first impact, indicating a clear intensification of damage during the second loading.

**Figure 8:**
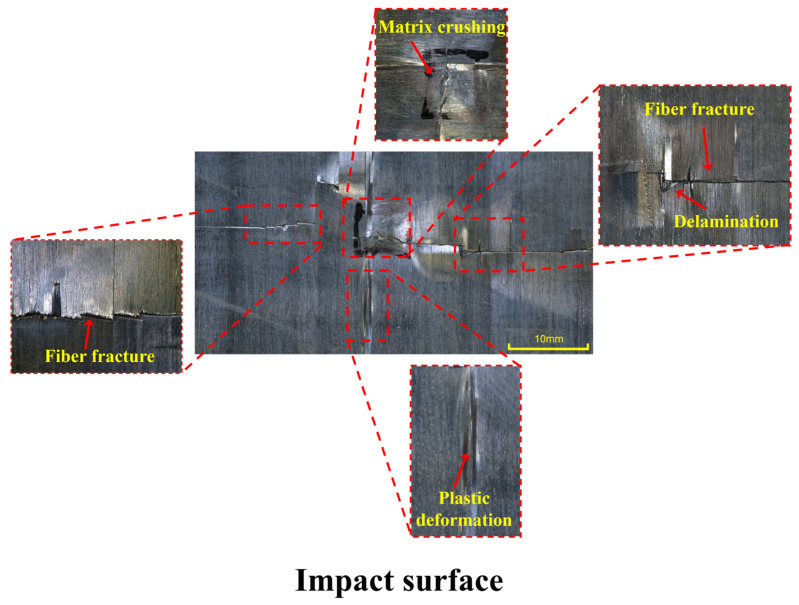
Typical damage morphology of CF/PEEK laminates after the second impact at 30 J: Impacted surface.

**Figure 9:**
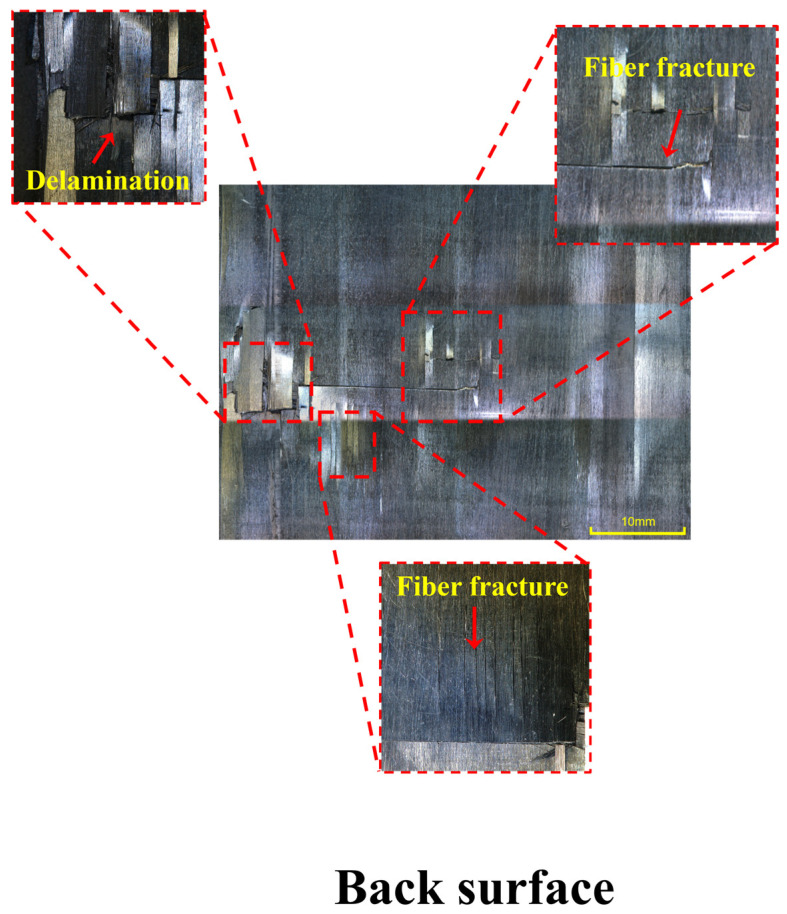
Typical damage morphology of CF/PEEK laminates after the second impact at 30 J: Back surface.

The damage morphology of the impacted and back surfaces of CF/PEEK laminates after the second impact at 30 J is shown in Figure 8 and Figure 9. Detailed annotations in the locally enlarged images reveal the typical damage mechanisms of the material under high-energy repeated impacts. From the impacted surface (Figure 8), extensive surface damage was observed, including matrix crushing, pronounced plastic deformation, repeated fiber fractures, and interlaminar delamination. The central matrix region exhibited signs of collapse and fragmentation, indicating that a high stress concentration had developed locally under the impact load, which led to matrix crushing failure. Irregular deformation boundaries surrounding the plastic zone reflected that the energy buffering capacity of the thermoplastic matrix was approaching its limit under the second impact.

More critically, fiber fractures and delamination were interwoven throughout the damaged area, indicating simultaneous failure of both the reinforcement phase and the interfacial regions, resulting in a significant reduction in structural load-bearing capacity. The back-side image (Figure 9) further revealed a through-thickness failure mode. Continuous surface cracks and exposed fiber fractures were observed at repeated locations, and the delamination boundaries were clearly visible. These features indicate that the impact wave had propagated through the laminate thickness, leading to a penetration failure characterized by surface matrix crushing on the front side and tearing on the back side. This type of failure typically reflects that the structural damage tolerance had been exceeded and that energy dissipation could no longer be achieved through local plastic deformation or interfacial delamination.

Overall, the impact and back-surface morphologies resulting from the second 30 J impact exhibited highly coupled multi-mechanism failure modes, including matrix crushing, fiber fracture, interfacial debonding, and through-thickness cracking. These combined effects indicate that the laminate had reached its ultimate failure state under high-energy repeated impacts. Compared with low-energy impacts, the failure mode at this stage had transitioned from “damage propagation” to “structural failure” [40].

### 3.3. Delamination Damage Analysis

In thermoplastic composite laminates, delamination is widely recognized as a dominant impact-induced failure mode that governs both load-bearing capacity and energy dissipation behavior. It is also a critical factor in assessing damage tolerance and predicting structural failure. To investigate the interfacial degradation of CF/PEEK laminates under repeated impact loading, delamination behavior was systematically characterized and analyzed.

**Figure 10:**
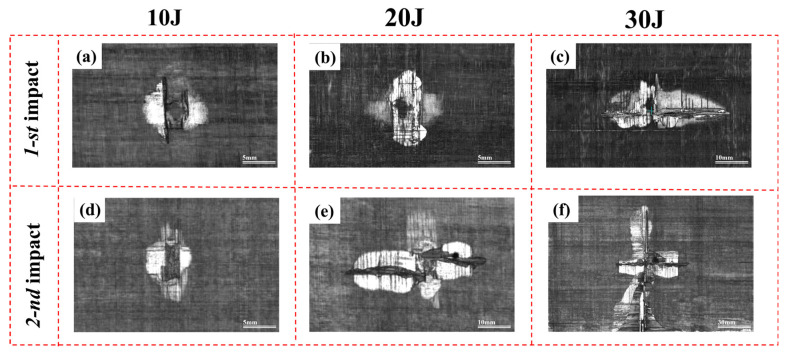
C-scan images of CF/PEEK laminates under different impact energies and repeated impacts: (**a**–**c**) delamination distribution after the first impact at 10 J, 20 J, and 30 J; (**d**–**f**) delamination distribution after the second impact at 10 J, 20 J, and 30 J.

The extent and distribution of delamination under varying impact energies and repeated impact conditions were evaluated using ultrasonic nondestructive inspection, as illustrated in Figure 10. Figure 10a–f presents the C-scan images of CF/PEEK laminates after single and repeated impacts. The delaminated areas are clearly identified through contrast variations in grayscale, revealing the geometry, propagation range, and potential penetration trends of interlaminar damage under different impact scenarios. Figure 10a–c corresponds to delamination patterns following the first impact, while Figure 10d–f shows the evolution of delamination at the same locations after the second impact. The impact energy increases progressively from left to right.

In the first impact series, Figure 10a,b show the delamination morphology under low (10 J) and moderate (20 J) impact energies, respectively. In Figure 10a, the delamination is relatively limited and oriented along the vertical direction centered around the impact point, forming a “dumbbell-shaped” symmetric pattern, indicating localized energy concentration. Figure 10b shows a significant increase in delaminated area, along with signs of interlaminar shear and symmetric lateral propagation. In Figure 10c, corresponding to a 30 J impact, the delamination area expands substantially and exhibits a “cross-shaped” morphology, indicating that the high-energy impact caused extensive interfacial separation across repeated ply interfaces, along with pronounced structural instability.

In comparison, the second impact results shown in Figure 10d–f demonstrate a more severe delamination expansion. Despite the impact energy remaining at 10 J in Figure 10d, the delamination area increased noticeably, showing an outward growth pattern suggestive of further propagation from the initial damage zone. In Figure 10e, the second impact at 20 J resulted in a blurred delamination boundary and asymmetric spread, likely caused by stress redistribution induced by pre-existing damage. Figure 10f shows the outcome of a second impact at 30 J, where delamination extended across nearly the entire laminate. A dark absorption region is visible at the impact center, indicating severe fiber–matrix damage and a dispersed multi-crack pattern. This reflects a through-thickness failure mode and suggests that the structure had undergone catastrophic failure under high-energy repeated impact.

To quantitatively evaluate the delamination growth behavior of CF/PEEK laminates under varying impact energies and repeated loading, the delaminated areas were identified and calculated based on C-scan images.

**Figure 11:**
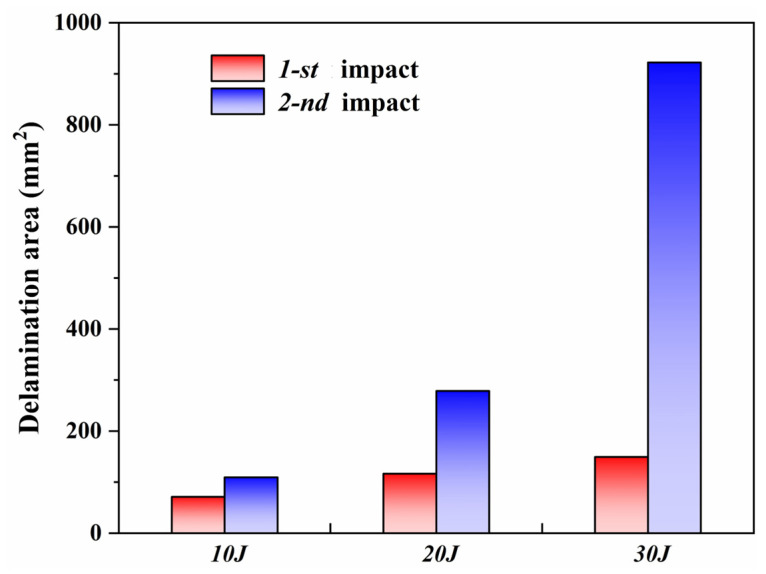
Comparison of delaminated areas in CF/PEEK laminates under different impact energies and repeated impacts.

The original C-scan images were converted to grayscale, followed by binary processing using a fixed threshold to extract the delaminated regions. The pixel-to-area conversion was calibrated according to the reference scale on the specimens, allowing the actual delamination area to be computed. As shown in Figure 11, with increasing impact energy, the delaminated area increased significantly after both the first and second impacts. Moreover, the delamination growth induced by the second impact was markedly greater than that caused by the first. This observation suggests that the localized damage induced by the initial impact facilitated further interlaminar failure during the subsequent impact. Particularly under high energy conditions, damage evolution was intensified, exhibiting a pronounced cumulative failure effect.

Overall, thermoplastic composites demonstrated typical progressive delamination behavior under repeated impacts, revealing clear energy sensitivity and damage evolution characteristics in their impact response.

During the first impact, the delamination area progressively increased from 71.68 mm^2^ to 149.64 mm^2^ as the impact energy rose from 10 J to 30 J, as shown in Table 3, demonstrating a typical energy-dependent damage pattern. After the second impact, the delamination behavior exhibited a more complex evolution. For the 10 J and 20 J cases, the delamination areas increased to 109.62 mm^2^ and 278.91 mm^2^, respectively, indicating further expansion based on the initial damage and reflecting a typical cumulative damage effect. However, under the 30 J condition, despite already being the highest energy level, the delamination area surged to 922.10 mm^2^ after the second impact, significantly exceeding the other cases. This suggests that under high-energy repeated impacts, local structural instability, fiber/matrix interfacial failure, and fracture progressed rapidly, leading to severe delamination propagation.

## 4. Conclusions

In order to investigate the load-bearing increase in thermoplastic composites under repeated impact loading, a systematic experimental and morphological study was conducted on CF/PEEK laminates. Low-velocity impact tests with varying energy levels were performed under both the first and second impacts. The mechanical responses were analyzed to assess load-bearing capacity and energy absorption behavior, while optical microscopy and ultrasonic C-scan techniques were used to evaluate surface damage and delamination propagation.

The CF/PEEK laminate exhibited favorable impact resistance under the first impact. During the second impact, the peak loads under 10 J and 20 J conditions increased by approximately 12.64% and 16.12%, respectively, indicating a certain degree of compaction and stiffness increase. This behavior results from the combined effects of plastic deformation, local compaction, and matrix deformation and damage. During the first impact, the absorbed energy increased significantly with rising impact energy. During the second impact, the absorbed energy decreased by approximately 11.6% (10 J) and 15.2% (20 J) compared to the first impact, indicating a marked decline in energy dissipation capacity. This reduction was primarily attributed to the plastic deformation, delamination propagation, and matrix damage induced by the initial impact, which weakened the ability to sustain energy absorption under repeated loading. During the first impact, load-bearing increase was observed in the force–displacement responses at different impact energy levels, whereas the initial impact also introduced damage, which caused more pronounced damage propagation under the subsequent impact. Compaction-hardening increases local contact stiffness and indentation resistance, which elevates peak load in the subsequent impact, while fibers remain the primary load-bearing constituents.

By systematically examining the front and back surface morphologies of the laminates after impact, it was found that the failure mode evolved from localized indentation and matrix cracking to distinct fiber fracture, matrix debonding, and even penetration failure. Compared to single impacts, more severe damage was observed after repeated impacts, where pre-existing cracks and delaminated regions further propagated both through the thickness and in-plane directions. Under the second impact, local structures exhibited severe damage features, including fragmentation and penetration failure.

For thermoplastic composites, the load-bearing increase in the matrix enhances the mechanical response under repeated impacts, while it also introduces matrix cracking, fiber damage, and delamination. Therefore, the impact-resistant structural design of thermoplastic composites should consider both structural design and the hardening effect.

## Figures and Tables

**Table 1:** Comparison of peak loads in the first and second impacts of CF/PEEK laminates.

Impact Energy (J)	Peak Load of *1-st* Impact (kN)	Peak Load of *2-nd* Impact (kN)	ΔPeak Load (kN)	Change Rate
10	3.203	3.608	0.405	12.64% ↑
20	4.898	5.687	0.789	16.12% ↑
30	6.671	7.216	0.545	8.17% ↑

**Table 2:** Comparison of absorbed energy under repeated impacts at different energy levels.

Impact Energy (J)	*1-st* Impact Absorbed Energy (J)	*2-nd* Impact Absorbed Energy (J)	ΔEnergy (J)	Change Rate
10	4.837	2.543	2.294	47.42% ↓
20	10.802	7.043	3.685	34.11% ↓
30	18.331	---	---	---

**Table 3:** Comparison of delamination areas under repeated impacts at different energy levels.

Impact Energy (J)	*1-st* Impact Delamination Area (mm^2^)	*2-nd* Impact Delamination Area (mm^2^)	Area Growth (mm^2^)
10	71.68	109.62	37.94
20	116.92	278.91	161.99
30	149.64	922.10	772.46

## Data Availability

The data that support the findings of this study are available from the corresponding author, Xu, upon reasonable request.
